# Exosomal MicroRNAs Derived from Human Amniotic Epithelial Cells Accelerate Wound Healing by Promoting the Proliferation and Migration of Fibroblasts

**DOI:** 10.1155/2018/5420463

**Published:** 2018-07-25

**Authors:** Bin Zhao, Xiaodong Li, Xiaomin Shi, Xueqin Shi, Wei Zhang, Gaofeng Wu, Xujie Wang, Linlin Su, Dahai Hu

**Affiliations:** ^1^Department of Burns and Cutaneous Surgery, Xijing Hospital, Fourth Military Medical University, Xi'an, Shannxi 710032, China; ^2^School of Life Sciences, Northwestern Polytechnical University, Xi'an, Shannxi 710072, China; ^3^Department of Burns and Plastic Surgery, General Hospital of Lanzhou Petrochemical Company, Lanzhou, Gansu 730060, China

## Abstract

Previous work in our laboratory demonstrated that exosomes derived from human amniotic epithelial cells (hAECs) accelerated wound healing by promoting the proliferation and migration of fibroblasts. It is reported that exosomes, which are carriers of the microRNAs (miRNAs) and proteins, play an important role in the regulation of cell-to-cell communication. However, it is still unclear precisely which molecule or which group of molecules carried within hAEC-derived exosomes (hAEC-Exos) mediated wound healing. Here, we explored purified hAEC-Exos together with either proteinase K (PROse) or RNase A on the effect of fibroblasts and cutaneous wound healing. Our experiments demonstrated that hAEC-Exos were positive for exosomal markers CD9, CD63, and CD81. Also, we found that hAEC-Exos could be internalized by fibroblasts and then stimulated cell migration and proliferation. However, the promotive effect of hAEC-Exos was abolished by pretreating hAEC-Exos with RNase A, not PROse. Importantly, *in vivo* wound healing assay showed that local injection of hAEC-Exos or PROse pretreated hAEC-Exos at skin wounds significantly accelerated wound healing. Our findings revealed an important role of exosomal miRNAs in wound healing.

## 1. Introduction

Wound healing is a dynamic physiological process which consists of fibroblast proliferation and extracellular matrix (ECM) remodelling [1]. During the proliferation phase, both cell proliferation and migration are required for the formation of granulation tissues that fill up the defective skin. At the phase of ECM remodelling, fibroblasts proliferate within the wounds and synthesize ECM such as collagen and fibronectin.

Recent studies demonstrated that stem cell therapy promoted wound healing mainly through a paracrine mechanism [2, 3], in which exosomes played a dominant role [4]. Exosomes, a kind of membrane lipid vesicle with a diameter of 30–150 nm, were reported to be an important mediator for cell-to-cell communication [5]. Accumulative evidences implicated that exosomes function in diverse biological processes [6].

Additionally, our previous work demonstrated that hAEC-Exos accelerated wound healing by promoting the proliferation and migration of fibroblasts [7]. However, the components within hAEC-Exos exerting key roles in skin wound healing are not clear. Here, we explored the effect of purified hAEC-Exos together with either PROse or RNase A on the proliferation and migration of fibroblasts. Both the *in vitro* and *in vivo* experiments showed that hAEC-Exos-derived miRNAs promoted the proliferation and migration of fibroblasts and further accelerated wound healing. These data presented strong evidence that hAEC-Exos-derived miRNAs would have promising clinical application on wound healing.

## 2. Methods

### 2.1. Patients and Ethical Approval

Human amnion tissues were obtained from five pregnant women (mean age of 26 years) in the Department of Obstetrics, Xijing Hospital (Xi'an, China). All experiments including humans and animals were conducted under the protocols reviewed and approved by the Xijing Hospital Ethics Committees. The written informed consents were obtained from all patients or their legal guardians.

### 2.2. *In Vivo* Wound Healing

Male BALB/c mice weighing 20–25 g were purchased from the Animal Center of Fourth Military Medical University and housed under standard laboratory conditions. Mice were randomly divided into four groups: (1) the phosphate buffer saline (PBS) control group (*n* = 5); (2) the Exos-RNase treatment group (*n* = 5); (3) the Exos-PROse treatment group (*n* = 5); and (4) the exosome treatment group (*n* = 5). Mice were anesthetized by intraperitoneal injection of pentobarbital sodium (20 mg/kg bw). The hair on the back and flank were clipped; 1 cm × 1 cm full-thickness wounds were generated on each mouse as described [8]. We previously reported that 50 *μ*g/mL exosomes derived from human amniotic epithelial cells accelerated wound healing [7]. Herein, 100 *μ*L of 50 *μ*g/mL exosomes or equal amount of exosomes together with either PROse or RNase A were injected into the surrounding tissues of the wounds at four sites on day 1 and day 3. In the control group, equal amount of PBS was injected. The wound size was measured and analyzed in a timely manner with the Image Pro Plus 6.0 software (Medical Cybernetics, USA) according to the protocol [9]. Skin tissue samples were collected for further histological analysis. Wound closure rate (%) was calculated as ((original wound area on day 0 − open area on day *X*)/original wound area on day 0) × 100%.

### 2.3. Histological Examination

Skin samples were harvested, fixed, dehydrated, paraffin-embedded, and then sliced into 4 *μ*m thick sections, followed by hematoxylin-eosin staining (H&E) and immunohistochemistry (IHC) as described previously [10, 11]. The images of stained sections were obtained by the FSX100 microscope (Olympus, Japan).

### 2.4. Isolation of hAECs and hAEC-Exos

hAECs were isolated and cultured as previously described [12]. Briefly, human amnion layer was mechanically peeled off from the placenta and rinsed with sterilized PBS. Amniotic membranes were subjected to 0.25% trypsin and incubated at 37°C with constant agitation for 1 h. Trypsin was inactivated by the addition of DMEM supplemented with 10% exosome-depleted FBS (System Biosciences, USA, number 022515). Collected hAECs were filtered through a 200 *μ*m cell strainer and centrifuged at 1000 ×g for 5 min; isolated hAECs were cultured in DMEM supplemented with 10% exosome-depleted FBS and 1% antibiotic-antimycotic in a humidified incubator at 37°C with 5% CO_2_.

For the isolation of hAEC-Exos, 1 × 10^6^ hAECs were seeded into the T75 Culture Flask (Corning, USA) in DMEM supplemented with 10% exosome-depleted FBS for 48 h; the culture medium of hAECs was collected and centrifuged at 300 ×g for 5 min. After centrifugation, the supernatant was collected and filtered through a 0.22 *μ*m filter (Millipore, USA) to remove cell debris. The remaining supernatant was then ultracentrifuged at 100,000 ×g with the rotor Ti70 (Beckman Coulter, USA) for 12 h. The exosome-enriched pellets were obtained and resuspended in a small volume of PBS and then measured for protein content using the BCA protein assay kit (BOSTER Biological Technology, China) and adjusted the concentration to 50 *μ*g/mL, stored at −80°C. hAEC-Exos were examined to confirm their characteristics with nanoparticle tracking analyzer, transmission electron microscope, flow cytometry, and western blot, respectively.

### 2.5. Size Distribution and Zeta-Potential of hAEC-Exos

The size and zeta-potential of exosomes were examined by the Nanoparticle Tracking Analyzer (Particle Metrix GmbH, Germany) with the corresponding ZetaView® software [13]. This instrument tracked the Brownian motion of particles over time and subsequently calculated particle sizes.

### 2.6. Transmission Electron Microscope (TEM)

For TEM detection, exosomes were fixed with 2% phosphotungstic acid, dropped onto a formvar/carbon-coated copper mesh grids, and then left to dry at room temperature. Exosomes were examined with a Hitachi 7100 transmission electron microscope (Hitachi, Japan).

### 2.7. Flow Cytometry

The exosomes were confirmed by exosomal surface marker CD63 using flow cytometry [14]. Briefly, exosomes were attached onto 4 *μ*m aldehyde/sulfate latex beads and incubated with exosome-CD63 antibody (System Biosciences, USA). The percent of positive beads was calculated by the FACSAria III instrument (BD, USA).

### 2.8. Cell Treatment

According to previous studies, exosomes mainly contain proteins and RNAs [15]. To determine which molecules carried within hAEC-Exos were responsible for wound healing, purified hAEC-Exos were pretreated with 0.04% Triton X-100 and then treated with RNase A (100 units/mL) for 60 min to deplete RNAs or PROse (100 *μ*g/mL) for 60 min to degrade exosomal proteins according to the protocol described previously [16, 17]. The efficacy of PROse or RNase enzyme treatments was detected by gel electrophoresis or Coomassie blue G250 staining, respectively. Furthermore, we also tested the protein concentration by the BCA protein assay kit (BOSTER Biological Technology, China) and RNA content by spectrophotometric ratio using absorbance measurements at wavelengths of 260 nm and 280 nm. Adult human dermal fibroblasts (ATCC, USA) were divided into four groups. Cells in experimental groups were treated with equal amounts of purified hAEC-Exos together with either PROse or RNase A. Equal amounts of hAEC-Exos treated with 0.04% Triton X-100 serve as control.

### 2.9. PKH26 Labeling of Exosomes

Exosomes were labeled with PKH26 (Sigma, Germany) according to the manufacturer's protocol. Briefly, exosomes were resuspended in diluent C (Sigma, Germany), mixed with PKH26 into a final concentration of 1 × 10^−6^ M, and then incubated at 37°C for 5 min. Excess dye was removed by centrifugation. PKH26-labeled exosomes were cocultured with fibroblasts in FBS-free medium for 12 h. The internalization of hAEC-Exos by fibroblasts was counterstained with 4′,6-diamidino-2-phenylindole (DAPI) (Sigma, Germany) and observed under the ZEISS inverted fluorescence microscope (ZEISS, Germany).

### 2.10. Immunofluorescent Staining

To detect the internalization of PKH26-labeled exosomes by fibroblasts, cells were fixed with 4% formaldehyde and washed with PBS. Nuclei were counterstained with DAPI (Sigma, Germany). Images were taken by using the FSX100 microscope (Olympus, Japan). Ki67 was stained and used for evaluating cell proliferation. Briefly, fibroblasts were seeded at a density of 1 × 10^4^ cells/cm^2^, incubated in serum-depleted medium for 12 h, stimulated with hAEC-Exos for another 48 h, and then incubated with anti-ki67 antibody (Abcam, USA) and counterstained with DAPI.

### 2.11. Western Blot

Exosomes were lysed in lysis buffer containing a complete protease inhibitor tablet (Roche, Swiss). Five micrograms of proteins was loaded onto polyacrylamide gel, separated by electrophoresis, and then transferred to the polyvinylidene difluoride membrane (PVDF). After blocking with 5% nonfat milk, PVDF membrane was incubated with rabbit monoclonal anti-human CD63, CD9, and CD81 antibodies (Abcam, USA) overnight at 4°C, followed by the incubation with horseradish peroxidase-conjugated goat anti-rabbit secondary antibody (BOSTER Biological Technology, China). Proteins were visualized by the enhanced chemiluminescence system (Alpha Innotech, USA).

### 2.12. Real-Time Cell Proliferation Analysis

Fibroblasts were seeded into 16 E-plates (ACEA, USA) at the density of 5 × 10^3^ cells/well and cultured in a humidified 5% CO_2_ incubator. To avoid the interference of background, in the first step of an RTCA assay, cell medium is added to the wells and a background value is taken, after that, different treatments were applied to each well for 24 h (*n* = 3). The cell index in each E-plate well was calculated by the RTCA software 1.2 (Roche, France). The graphs were real-time outputs generated from the iCELLigence system (ACEA, USA) [18].

### 2.13. Migration Assay

Fibroblasts were subjected to different treatments in conventional scratch wound assay [19]. Briefly, fibroblasts were seeded at a density of 1 × 10^5^ cells in a 35 mm dish and starved in serum-free DMEM for 12 h. Gaps were created in the middle of each well in cross shape with a pipette tip. Cells were then washed gently with PBS and applied with different treatments, followed by incubation at 37°C in 5% CO_2_ in air atmosphere for 24 h. Images were taken in a timely manner. Gap areas were measured and recorded and then compared to the initial gap size at 0 h by the Image Pro Plus 6.0 software. The area of migration was calculated as (%) = ((original blank area − blank area on *X* h)/original blank area) × 100%. The migration of fibroblasts was also determined by transwell assay using 8 *μ*m pore filters according to the manufacturer's recommendation. Approximately 1 × 10^5^ fibroblasts were seeded into the upper compartment, while exosomes with different treatments were added into the lower compartment. Cells were cocultured for 24 h; nonmigrated cells in the upper chamber were wiped and stained with 0.4% crystal violet.

### 2.14. Statistical Analysis

All data were presented as the mean ± standard deviation (SD). Statistical analyses were performed using the SPSS 11.0 software (SPSS Inc., USA). One-way ANOVA was used to compare among groups, followed by the Bonferroni post hoc test. *P* < 0.05 was considered statistically significant.

## 3. Results

### 3.1. Isolation and Characterization of hAEC-Exos

**Figure 1:**
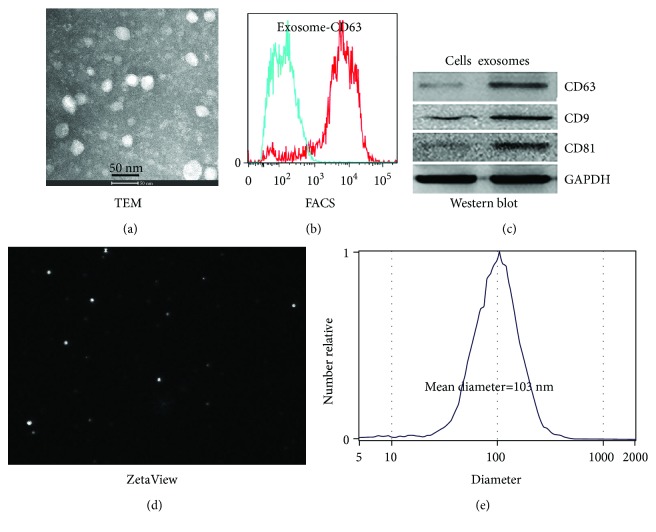
Characterization of hAEC-Exos. (a) TEM image of hAEC-Exos (*n* = 5). (b) Flow cytometry analysis of exosomal marker CD63 (*n* = 4). (c) Western blot analysis of exosomal markers CD63, CD9, and CD81 (*n* = 3). (d) ZetaView analysis of hAEC-Exos (*n* = 3). (e) Size distribution of hAEC-Exos (mean diameter = 103 nm, *n* = 3).

Exosomes with round or oval morphology were observed by TEM; the diameters ranged from 30 to 150 nm (Figure 1(a)). Flow cytometry was used to analyze exosomal surface marker CD63, and results showed that hAEC-Exos were positive for CD63 (85.2% ± 4.8%) (Figure 1(b)). Western blot results further confirmed that exosomal markers CD9, CD63, and CD81 were expressed in hAEC-Exos (Figure 1(c)). Dynamic tracking capture (Figure 1(d)) and particle size distribution (Figure 1(e)) were measured by nanoparticle tracking analyzer; results showed that the size of 90% particles distributed between 30 and 150 nm (the average diameter = 103 nm).

### 3.2. Evaluation of hAEC-Exos Together with Either PROse Or RNase A

**Figure 2:**
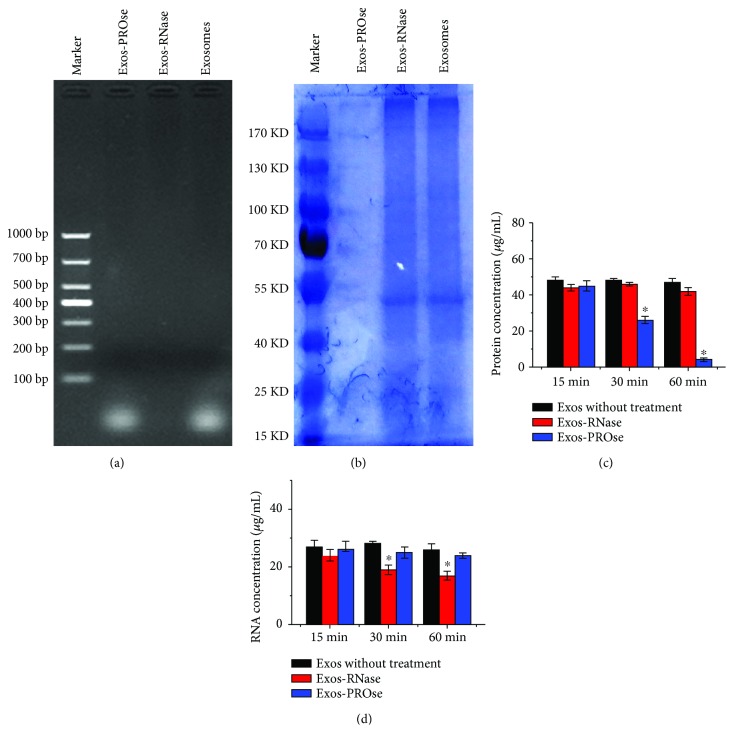
Characterization of purified hAEC-Exos together with either PROse or RNase treatment. (a) Gel electrophores show that exosomes were depleted of RNAs after RNase digestion. (b) Coomassie blue staining shows that proteins were thoroughly degraded after PROse treatment (*n* = 3). (c) Detection of protein concentration by the BCA protein assay kit and RNA content (d) by the spectrophotometric ratio (^∗^ *P* < 0.05, *n* = 3).

Purified hAEC-Exos were pretreated with 0.04% Triton X-100 and then treated with PROse or RNase A. Results from gel electrophoresis (Figure 2(a)) showed that RNA components carried within hAEC-Exos were mainly small RNAs with the size below 100 base-pair, while protein components in hAEC-Exos were almost completely degraded by PROse treatment (Figure 2(b)). Furthermore, we tested the protein concentration (Figure 2(c)) by the BCA protein assay kit and RNA content (Figure 2(d)) by the spectrophotometric ratio using absorbance measurements at wavelengths of 260 nm and 280 nm. As shown in Figure 2(c), after 60 min of PROse incubation, the proteins contained in hAEC-Exos were barely detectable. However, the RNase-treated hAEC-Exos still maintained RNA as detected (Figure 2(d)). In addition, the gel electrophoresis results showed that RNA components carried within hAEC-Exos were mainly small RNAs with the size below 100 base-pair (Figure 2(a)). Therefore, these results demonstrated that the RNAs contained in RNase-treated exosomes might be miRNAs.

### 3.3. RNA Components of hAEC-Exos Promote the Proliferation of Fibroblasts

**Figure 3:**
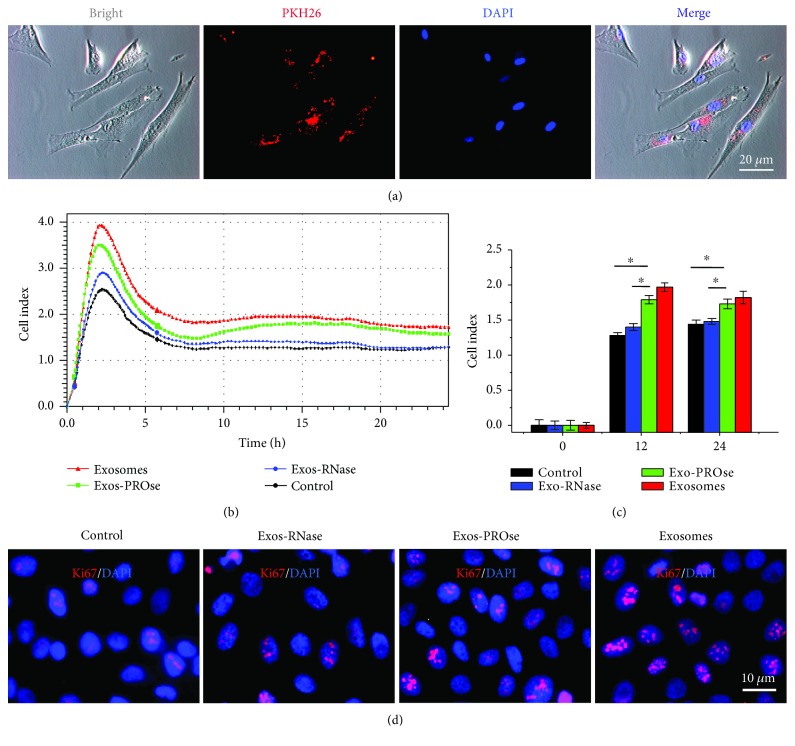
Effects of hAEC-Exos on the proliferation of fibroblasts. (a) Fluorescent microscopy analysis shows that PKH26-labeled hAEC-Exos were internalized by fibroblasts. Nuclei were counterstained with DAPI. Scale bar = 20 *μ*m. (b) The evaluation of fibroblast proliferation after different hAEC-Exos treatments by the iCELLigence system. Each group was assessed in triplicate. Fibroblasts without any treatment serve as control. (c) Quantitative analysis of fibroblast proliferation at 12 h and 24 h. (d) Fluorescent microscopy analysis of ki67 immunostaining (red). Nuclei were counterstained with DAPI. Scale bar = 10 *μ*m. (^∗^ *P* < 0.05, *n* = 3).

In *in vitro* tracking experiment, PKH26-labeled exosomes were incubated with fibroblasts for 24 h; the cellular uptake of hAEC-Exos was evaluated via the ZEISS fluorescence microscopy (Figure 3(a)). We observed the presence of PKH26-positive granules in the perinuclear region of fibroblasts by the ZEISS inverted fluorescence microscope, suggesting that hAEC-Exos could be internalized by fibroblasts. To determine which component in hAEC-Exos exerted the positive effect on fibroblasts, we individually added PROse-treated or RNase-treated hAEC-Exos into fibroblasts. Fibroblasts without any treatment serve as control. As shown in Figure 3(b), the presence of hAEC-Exos or PROse-treated hAEC-Exos significantly increased cell index value for the whole duration. However, RNase-treated hAEC-Exos lost the ability to promote the proliferation of fibroblasts. The immunostaining of ki67, a cell proliferation marker [20], also confirmed the above changes (Figure 3(d)). Results showed that the number of ki67-positive nuclei in PROse-treated group was more than that in RNase-treated group. In *in vivo* study, we injected PKH26-labeled exosomes into skin wounds, and vimentin, the marker of fibroblasts, was also costained. The distribution of PKH26-labeled exosomes was observed by the ZEISS inverted fluorescence microscope after frozen section. As shown in Figure S1, we can clearly see the costained exosomes and fibroblasts (orange) on days 1, 3, and 5 postinjection, indicating that injected PKH26-labeled exosomes may be internalized by fibroblast *in vivo*.

### 3.4. RNA Components of hAEC-Exos Promote the Migration of Fibroblasts

**Figure 4:**
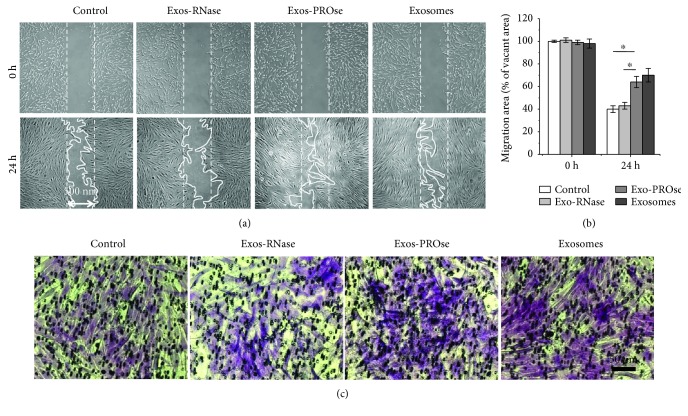
Effects of hAEC-Exos on the migration of fibroblasts. (a) Representative images of the scratch wound assay treated by hAEC-Exos at different time points. (b) Quantitative analysis of fibroblast migration. (c) Microscopic view of migrated cells after crystal violet staining. Scale bar = 50 *μ*m (^∗^ *P* < 0.05, *n* = 3).

Scratch wound assay (Figure 4(a)) and transwell assay (Figure 4(c)) were used to determine the effect of hAEC-Exos on the migration of fibroblasts. As shown in Figure 4(b), compared to the control or RNase-treated group, PROse-treated hAEC-Exos remarkably enhanced the migration of fibroblasts towards the blank area after 24 h treatment. Transwell assay obtained the similar results that hAEC-Exos and PROse-treated hAEC-Exos significantly enhanced the migration of fibroblasts compared to the control and RNase-treated hAEC-Exos. No obvious differences were observed between the RNase-treated hAEC-Exos group and the control group. Taken together, these results indicated that RNA components within hAEC-Exos played a key role in accelerating the migration of fibroblasts.

### 3.5. RNA Components of hAEC-Exos Promote Skin Wound Healing

**Figure 5:**
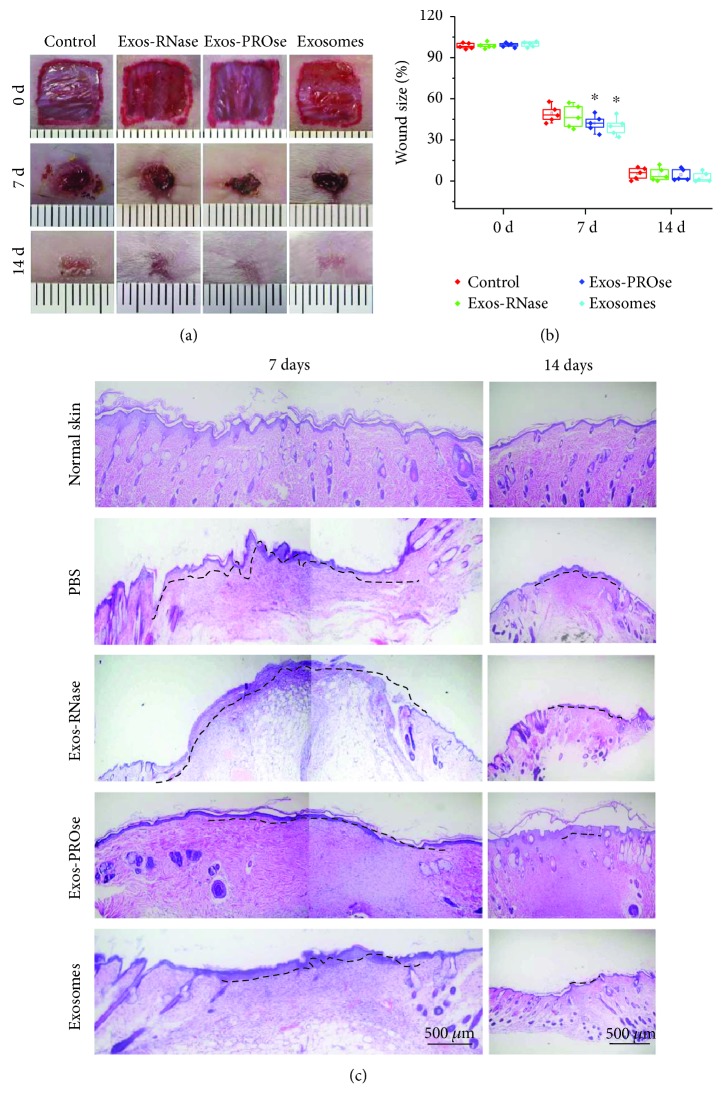
Wound closure assay on mice treated with hAEC-Exos. (a) Representative photographs of full-thickness excisional wounds treated with PBS, hAEC-Exos, Exos-PROse, or Exos-RNase. (b) Quantitative analysis of wound healing in each group (^∗^ *P* < 0.05, *n* = 5). (c) H&E staining of wounded skin sections in different groups on days 7 and 14 postwounding. Scale bar = 500 *μ*m (*n* = 5).

To further confirm the above results *in vivo*, excisional wounds (Figure 5(a)) on mice were treated with purified hAEC-Exos together with either PROse or RNase A via subcutaneous injection; injection of equal amount of PBS served as control. The median value of wound size (Figure 5(b)) showed that PROse-treated hAEC-Exos treatment markedly promoted the wound closure compared to the PBS control or the RNase-treated hAEC-Exos group on day 7 postwounding (*P* < 0.05). In addition, H&E staining was further evaluated to assess the quality of wound healing; as shown in Figure 5(c), PROse-treated hAEC-Exos markedly accelerated wound closure compared to the PBS control and the RNase-treated hAEC-Exos on day 7 postwounding. After 14 days, all wounds were completely healed; however, PROse-treated hAEC-Exos groups and hAEC-Exos groups seemed to be flatted compared to the control group or the RNase-treated hAEC-Exos group.

### 3.6. RNA Components of hAEC-Exos Ameliorate the Arrangement of Collagen Fibers

**Figure 6:**
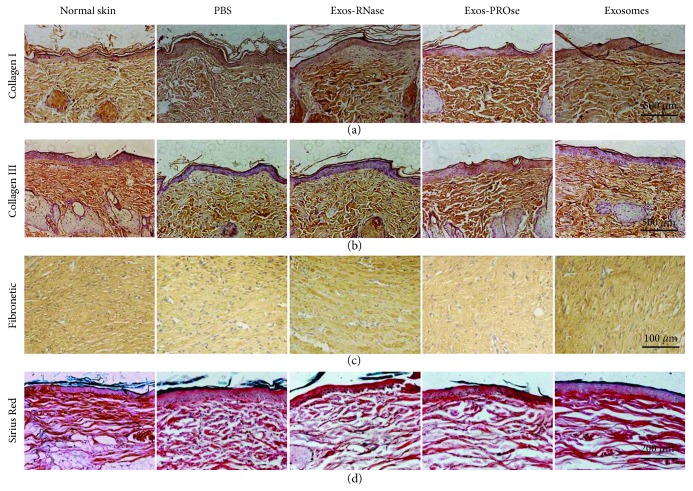
Collagen arrangement after wound healing. The IHC of collagen-I (a), collagen-III (b), and fibronectin (c) in wounded skin sections on day 30 postwounding. Scale bar = 500 *μ*m or Scale bar = 100 *μ*m (*n* = 5). (d) Sirius Red staining of collagen fibers on day 30 postwounding. Scale bar = 200 *μ*m (*n* = 5).

It is known that the remodelling of newly deposited ECM plays a major role in the process of hypertrophic scarring or skin fibrosis after wound healing [21]. We then evaluated the quality of wound healing by IHC on day 30 postwounding. The IHC of collagen-I (Figure 6(a)) and collagen-III (Figure 6(b)) showed that Exos-PROse-treated wounds presented the same well-reorganized collagen fibers as exosome-treated wounds. Moreover, the IHC of fibronectin in Exos-PROse-treated wounds presented as much as exosome-treated wounds (Figure 6(c)). Similar to the IHC results, Sirius Red staining (Figure 6(d)) also showed that RNA components of hAEC-Exos ameliorated the arrangement of collagen fibers, indicating that RNA components of hAEC-Exos not only promoted wound healing but also ameliorated the quality of skin wounds.

## 4. Discussion

hAECs, which can be obtained from the discarded amnion tissue via noninvasive procedure, have emerged as an alternative resource for stem cell therapy [22]. It was reported that the reparative effect of stem cells was mediated by paracrine signaling with the release of biologically active molecules affecting cell migration and/or proliferation [23]. In particular, the exosomes, an essential paracrine factor secreted by many cell types, played a dominant role in the regulation of tissue repair and regeneration [24]. Exosomes have been reported to “horizontally” transfer functional proteins or miRNAs to neighboring cells and thus serve as mediators of intercellular communication [25, 26]. Our previous study found that fluorescence-labeled hAEC-Exos were taken up by fibroblasts and enhanced the growth and migration of fibroblasts [7]. These findings were in line with the report that human fibrocyte-derived exosomes accelerate wound healing in genetically diabetic mice [27]. Likewise, Zhang et al. [28] reported that exosomes derived from human endothelial progenitor cells accelerate cutaneous wound healing by promoting angiogenesis through erk1/2 signaling. More recently, Zhao et al. [29] reported that exosomes derived from human umbilical cord mesenchymal stem cells relieved acute myocardial ischemic injury. In addition, Hu et al. [30] reported that exosomes derived from human adipose mesenchymal stem cells accelerated cutaneous wound healing via optimizing the characteristics of fibroblasts. However, the molecules within exosomes that mediated wound healing are still unclear. We further used PROse or RNase to determine which factors in hAEC-Exos could efficiently promote the proliferation and migration of fibroblasts, finding that hAEC-Exos-derived miRNAs would promote cell proliferation and migration. Our *in vivo* study further confirmed that hAEC-Exos-derived miRNAs ameliorated skin wounds and induced well-reorganized collagen fibers after wound healing. Similar observations were also reported by Xiao et al. [31] who found that exosomal miRNAs derived from amniotic fluid stem cells preserved ovarian follicles after chemotherapy. As well, Wang et al. [32] found that exosomes from induced pluripotent stem cells delivered cardioprotective miRNAs and prevented cardiomyocyte apoptosis in the ischemic myocardium. Furthermore, Fang et al. [16] confirmed that exosomal miRNAs from umbilical cord-derived mesenchymal stem cells suppressed myofibroblast differentiation by inhibiting TGF-*β*/Smad2 pathway during wound healing. It has been widely accepted that miRNAs interact with their mRNA targets by forming Watson-Crick pairings at their 5′ ends primarily with the 3′ UTRs of target mRNAs [33]. Therefore, we speculated that hAEC-Exos-derived miRNA may target its mRNAs and then accelerate wound healing by promoting the proliferation and migration of fibroblasts.

In this particular study, our findings led to important implications that stem cell-derived exosomal miRNA might act as a potential strategy for wound healing *in vivo*. Compared to other strategies, the exosome-based approach might be more safe and efficient, as it simulated the endogenous mechanism for cell–cell communications. In conclusion, the hAEC-Exos-based therapy could be a candidate strategy for promoting healing in the future. However, the precise mechanism through which miRNAs contribute to wound healing requires further investigation.

## Data Availability

The data used to support the findings of this study are available from the corresponding author upon request.

## Abbreviations

hAECs: Human amniotic epithelial cells
miRNAs: MicroRNAs
Exos: Exosomes
hAEC-Exos: Exosomes derived from human amniotic epithelial cells
TEM: Transmission electron microscope
ECM: Extracellular matrix
PBS: Phosphate buffer saline
H&E: Hematoxylin-eosin
IHC: Immunohistochemistry
DAPI: 4′,6-Diamidino-2-phenylindole
PVDF: Polyvinylidene difluoride membrane
PROse: Proteinase K.

## Supplementary Materials

Supplementary Materials Figure S1: costaining of exosomes and fibroblasts on wounds. Exosomes were labeled with PKH26 (red); fibroblasts were stained with vimentin. Scale bar = 100 *μ*m (*n* = 3).
