# Correction to “Exosomal MicroRNAs Derived from Human Amniotic Epithelial Cells Accelerate Wound Healing by Promoting the Proliferation and Migration of Fibroblasts”

**DOI:** 10.1155/sci/9874279

**Published:** 2026-02-18

B. Zhao, X. Li, X. Shi, et al., “Exosomal MicroRNAs Derived from Human Amniotic Epithelial Cells Accelerate Wound Healing by Promoting the Proliferation and Migration of Fibroblasts,” *Stem Cells International* 2018, no. 1 (2018): 5420463, https://doi.org/10.1155/2018/5420463.

In the article titled “Exosomal MicroRNAs Derived from Human Amniotic Epithelial Cells Accelerate Wound Healing by Promoting the Proliferation and Migration of Fibroblasts,” there is an error in Figure 6. More specifically, in Figure 6a,b, the representative image of IHC for Collagen I and Collagen III in the normal skin group shows tissue samples taken from rats instead of mice. In addition, the representative images of IHC for fibronectin in Figure 6c are all incorrect. The error occurred during figure assembly and the correct Figure 6 is shown below:

Figure 6Collagen arrangement after wound healing. The IHC of Collagen‐I (a), Collagen‐III (b), and fibronectin (c) in wounded skin sections on day 30 postwounding. Scale bars = 500 μm (*n* = 5). (d) Sirius red staining of collagen fibers on day 30 postwounding. Scale bar = 200 μm (*n* = 5).

**Figure:**
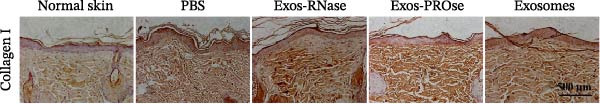
(a)

**Figure:**
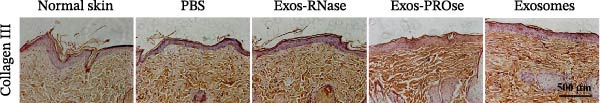
(b)

**Figure:**
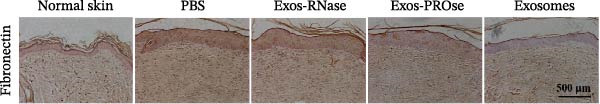
(c)

**Figure:**
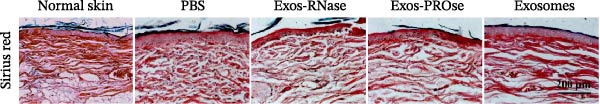
(d)

Furthermore, in Section 2.10. Immunofluorescent Staining, the text “Images were taken by using the FSX100 microscope (Olympus, Japan).” was incorrect. This should read: “Images were taken by using the Carl Zeiss Axio Observer A1 inverted fluorescence microscope.”

We apologize for these errors.

