# Supplementary figures and images for: Exosomal MicroRNAs Derived from Human Amniotic Epithelial Cells Accelerate Wound Healing by Promoting the Proliferation and Migration of Fibroblasts
Source: Stem Cells Int. 2018 Jul 25;2018:5420463. doi: 10.1155/2018/5420463 (PMC6083635; doi:10.1155/2018/5420463)

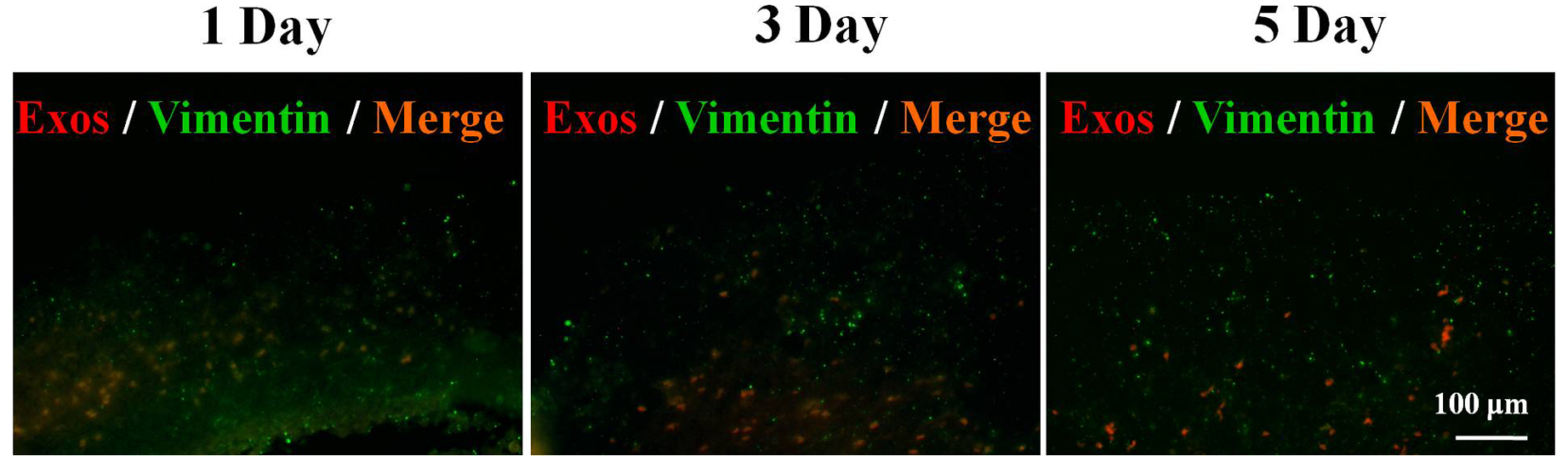

Supplement: Supplementary Materials — Figure S1: costaining of exosomes and fibroblasts on wounds. Exosomes were labeled with PKH26 (red); fibroblasts were stained with vimentin. Scale bar = 100 μm (n = 3). [file 5420463.f1.tif]
